# Supplementary material for: Listeria monocytogenes faecal carriage is common and depends on the gut microbiota
Source: Nat Commun. 2021 Nov 24;12:6826. doi: 10.1038/s41467-021-27069-y (PMC8613254; doi:10.1038/s41467-021-27069-y)
Supplement: Supplementary file 1 — Supplementary Information [file 41467_2021_27069_MOESM1_ESM.pdf]

Listeria monocytogenes faecal carriage is common and depends on the gut microbiota

Lukas Hafner[1,#], Maxime Pichon[2,3,#,§], Christophe Burucoa[2,3,§], Sophie HA Nusser[1], Alexandra Moura[1,4], Marc Garcia-Garcera[5,†], Marc Lecuit[1,4,6,†,*]

[1] Institut Pasteur, Université de Paris, Inserm U1117, Biology of Infection Unit, 75015 Paris, France
[2] University Hospital of Poitiers, Infectious Agents Department, Bacteriology and Infection Control Laboratory, 86021 Poitiers, France
[3] Université de Poitiers, Faculté de Médecine et de Pharmacie, EA 4331, 86022 Poitiers, France
[4] Institut Pasteur, National Reference Center and WHO Collaborating Center Listeria, 75015 Paris, France
[5] University of Lausanne, Department of Fundamental Microbiology, 1015 Lausanne, Switzerland
[6] Necker-Enfants Malades University Hospital, Division of Infectious Diseases and Tropical Medicine, APHP, Institut Imagine, 75006, Paris, France

# These authors contributed equally
† These authors jointly supervised this work
§ Current address: Université de Poitiers, Faculté de Médecine et de Pharmacie, Inserm U1070, 86022 Poitiers, France
* Correspondence: marc.lecuit@pasteur.fr

Supplementary Tables

Supplementary Table 1. Sensitivity of *hly* PCR in different matrices

Supplementary Table 2. Metadata of stool collection cohort

Supplementary Table 1: Sensitivity of *hly* PCR in different matrices

	-Stool sample		+Stool sample	
CFU	SB	EB	FSB	FEB
1×10^8	3/3	3/3	3/3	3/3
1×10^7	3/3	3/3	2/3	2/3
1×10^6	3/3	3/3	2/3	2/3
1×10^5	3/3	3/3	3/3	1/3
1×10^4	3/3	2/3	1/3	0/3
1×10^3	3/3	0/3	1/3	0/3
1×10^2	3/3	0/3	0/3	1/3
1×10^1	0/3	0/3	0/3	0/3

SB: Saline buffer, EB: eNat buffer, FSB: Negative stool sample diluted in saline buffer, FEB: Negative stool sample diluted in eNat buffer. The indicated amounts of *Lm* were resuspended in each matrix and a PCR of the *hly* gene was performed three times per sample. Red indicates samples that would be considered as negative with less than two positive PCR results, green indicates samples that would be considered as positive with at least two positive PCR results.

Supplementary Table 2: Metadata of stool collection cohort

	Lm-positive (n=90)	*Lm*-negative (n=810)	*P*-value
Median age (year; [IQR])	47.7 [38.2-60.7]	52.5 [39.9-63.6]	0.1812
Median delay before reception of the stool (day; [IQR])	1.00 (1-2)	1.00 (1-2)	0.5255
Male, n (%)	33 (36.7)	340 (42.0)	0.3677
Female, n (%)	57 (63.3)	470 (58.0)	
Birthplace			
Europe	85 (10.4)	730 (89.6)	0.2524
Africa	3 (1.4)	66 (98.6)	0.1407
Asia	0 (0)	8(100)	1.00
America	2 (25)	6 (75)	0.2079

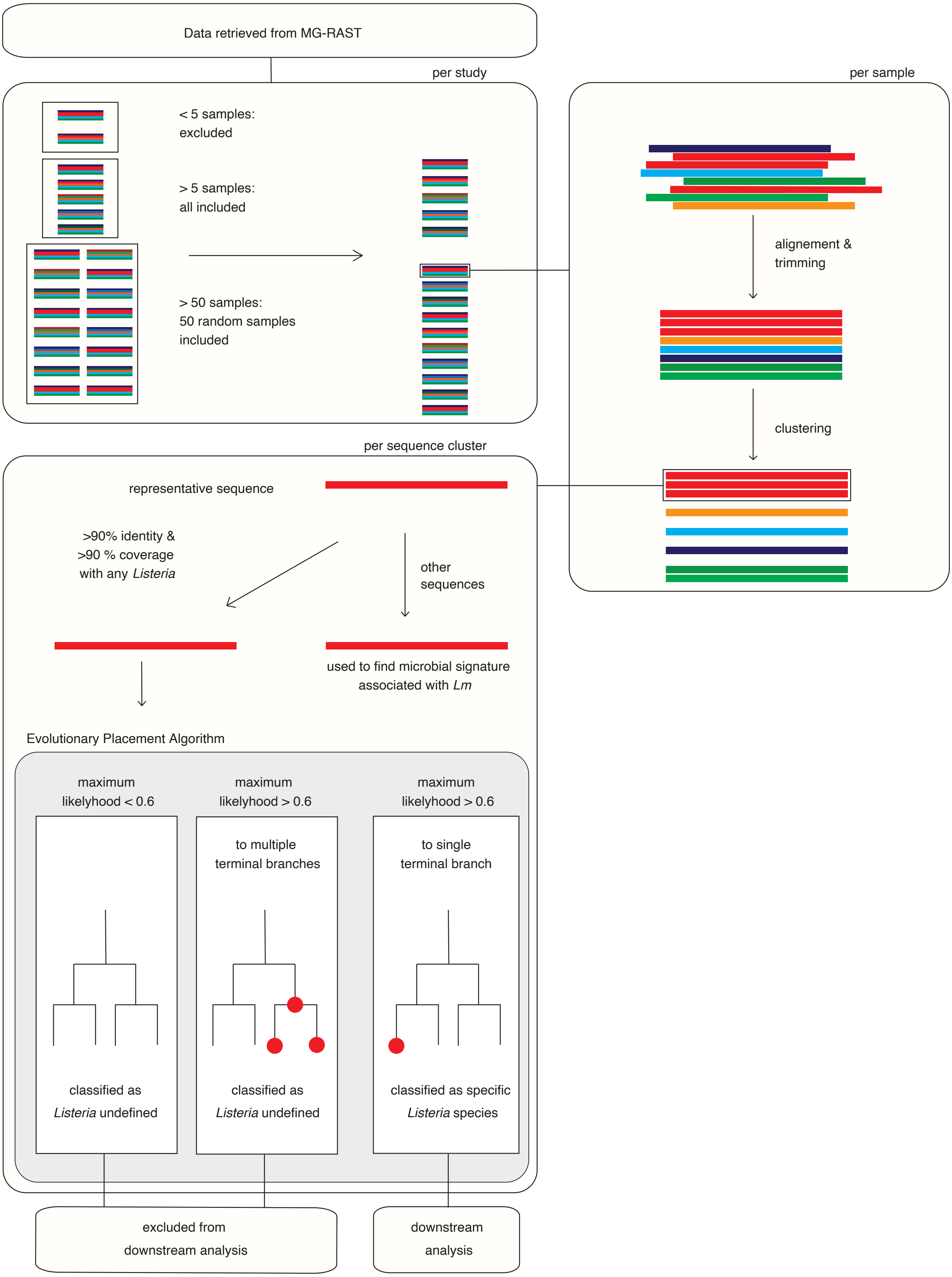

Supplementary Figure 1. Workflow of screening of *Listeria sp.* in 16S rRNA gene datasets

(Related to Material and Methods)

Data was retrieved from MG-RAST. Samples from studies with <5 samples were excluded, samples from studies with >5 and <50 samples and random selections of samples from studies with >50 samples were included. For each sample, sequences were aligned and trimmed, and then clustered. A representative sequence from each sequence cluster was then mapped to *Listeria sp.* sequences. Sequences with >90% identity and coverage with at least one *Listeria* were assigned to a tree of *Listeria* reference sequences by evolutionary placement algorithm. Sequences with a maximum likelihood >0.6 for a single terminal branch were assigned to the corresponding species, others were excluded from downstream analysis.
