# Supplementary material for: Listeria monocytogenes faecal carriage is common and depends on the gut microbiota
Source: Nat Commun. 2021 Nov 24;12:6826. doi: 10.1038/s41467-021-27069-y (PMC8613254; doi:10.1038/s41467-021-27069-y)
Supplement: Supplementary file 3 — Description of Additional Supplementary Files [file 41467_2021_27069_MOESM3_ESM.docx]

File Name: Supplementary Data 1.

Description: Sequenced PCR products from 10 *Lm-*positive human stool samples and EGD-e *hly* aligned by clustal omega.

File Name: Supplementary Data 2.

Description: Correlations of *Lm* abundance with microbial phyla. *P-*values were corrected for multiple testing

File Name: Supplementary Data 3.

Description: Correlations of *Lm* abundance with microbial families and orders. *P-*values were corrected for multiple testing

File Name: Supplementary Data 4.

Description: List of 16S rRNA datasets from MG-RAST used in this study

File Name: Supplementary Data 5.

Description: List of hosts analyzed in this study

File Name: Supplementary Data 6.

Description: List of full metagenome datasets from MG-RAST used in this study
