# Supplementary material for: Listeria monocytogenes faecal carriage is common and depends on the gut microbiota
Source: Nat Commun. 2021 Nov 24;12:6826. doi: 10.1038/s41467-021-27069-y (PMC8613254; doi:10.1038/s41467-021-27069-y)
Supplement: Supplementary file 10 — Reporting Summary [file 41467_2021_27069_MOESM10_ESM.pdf]

nature portfolio

Corresponding author(s): Marc Lecuit

Last updated by author(s): Oct 27, 2021

Reporting Summary

Nature Portfolio wishes to improve the reproducibility of the work that we publish. This form provides structure for consistency and transparency in reporting. For further information on Nature Portfolio policies, see our Editorial Policies and the Editorial Policy Checklist.

Statistics

For all statistical analyses, confirm that the following items are present in the figure legend, table legend, main text, or Methods section.

n/a	Confirmed	
☐	☒	The exact sample size (n) for each experimental group/condition, given as a discrete number and unit of measurement
☒	☐	A statement on whether measurements were taken from distinct samples or whether the same sample was measured repeatedly
☐	☒	The statistical test(s) used AND whether they are one- or two-sided<br>*Only common tests should be described solely by name; describe more complex techniques in the Methods section.*
☐	☒	A description of all covariates tested
☐	☒	A description of any assumptions or corrections, such as tests of normality and adjustment for multiple comparisons
☐	☒	A full description of the statistical parameters including central tendency (e.g. means) or other basic estimates (e.g. regression coefficient) AND variation (e.g. standard deviation) or associated estimates of uncertainty (e.g. confidence intervals)
☐	☒	For null hypothesis testing, the test statistic (e.g. F, t, r) with confidence intervals, effect sizes, degrees of freedom and P value noted<br>*Give P values as exact values whenever suitable.*
☒	☐	For Bayesian analysis, information on the choice of priors and Markov chain Monte Carlo settings
☒	☐	For hierarchical and complex designs, identification of the appropriate level for tests and full reporting of outcomes
☒	☐	Estimates of effect sizes (e.g. Cohen's d, Pearson's r), indicating how they were calculated

Our web collection on statistics for biologists contains articles on many of the points above.

Software and code

Policy information about availability of computer code

Data collection	See Methods
Data analysis	See Methods.<br>We used the following already published software/packages:<br>phyloseq v.1.34.0 (R package)<br>DESeq2 v.1.30.1 (R package)<br>micca v.1.7.2<br>IQ-tree v.1.6.5<br>usearch v.10.0.240<br>trimal v.1.4<br>mafft v. 7.407<br>SSU-align v.1.01

For manuscripts utilizing custom algorithms or software that are central to the research but not yet described in published literature, software must be made available to editors and reviewers. We strongly encourage code deposition in a community repository (e.g. GitHub). See the Nature Portfolio guidelines for submitting code & software for further information.

Data

Policy information about availability of data

All manuscripts must include a data availability statement. This statement should provide the following information, where applicable:

- Accession codes, unique identifiers, or web links for publicly available datasets
- A description of any restrictions on data availability
- For clinical datasets or third party data, please ensure that the statement adheres to our policy

We state: "Primary sequencing data are available on the Sequence Read Archive under the entry PRJNA642013. 16S rRNA datasets for screening of Listeria sp. were retrieved from MG-RAST."

Field-specific reporting

Please select the one below that is the best fit for your research. If you are not sure, read the appropriate sections before making your selection.

- [x] Life sciences
- [ ] Behavioural & social sciences
- [ ] Ecological, evolutionary & environmental sciences

For a reference copy of the document with all sections, see nature.com/documents/nr-reporting-summary-flat.pdf

Life sciences study design

All studies must disclose on these points even when the disclosure is negative.

Sample size	For mice experiments with control groups (e.g. Fig3f and Supplementary Fig 4), sample sizes were determined to minimize number of mice used and maximize statistical power.
Data exclusions	No data has been excluded.
Replication	All in vivo experiments have been replicated at least twice. PCRs to determine Lm in human feces were done in 3 individual replicates.
Randomization	We did not randomize mice during experiments. For human stool samples, randomization was not relevant as there was no control and treatment group.
Blinding	We did not apply blinding.

Reporting for specific materials, systems and methods

We require information from authors about some types of materials, experimental systems and methods used in many studies. Here, indicate whether each material, system or method listed is relevant to your study. If you are not sure if a list item applies to your research, read the appropriate section before selecting a response.

Materials & experimental systems

n/a	Involved in the study	
x		Antibodies
x		Eukaryotic cell lines
x		Palaeontology and archaeology
	x	Animals and other organisms
	x	Human research participants
x		Clinical data
x		Dual use research of concern

Methods

n/a	Involved in the study	
x		ChIP-seq
x		Flow cytometry
x		MRI-based neuroimaging

Animals and other organisms

Policy information about studies involving animals; ARRIVE guidelines recommended for reporting animal research

Laboratory animals	5-11 week old male and female mice, according to experiments (see Methods). Details on housing have been added to the manuscript.
Wild animals	No wild animals involved
Field-collected samples	Not applicable
Ethics oversight	All procedures were approved by the Animal Ethics Committe of Institut Pasteur (dap170057), authorized by the French Ministry of

Research under the reference number APAFIS#14655-2018041116183944 v1.

Note that full information on the approval of the study protocol must also be provided in the manuscript.

Human research participants

Policy information about studies involving human research participants

Population characteristics	See supplementary table S2
Recruitment	As in Pichon et al 2019: Consecutive adult patients without diarrhea referred for endoscopy and gastric biopsy on two random days per week were screened for inclusion as representative targets. Inclusion criteria were an age of ≥18years, referral as a hospitalized patient or an outpatient to one of the participating centers for endoscopy because of suspected H. pylori infection, a willingness to participate, and signing of an informed consent. Exclusion criteria were an age of <18years, recent or ongoing antibiotic treatment for ≤4weeks, previous receipt of eradication treatment, no affiliation with social insurance, receipt of legal protection, refusal to sign informed consent, inclusion in another trial, an inability to take oral medication on an ongoing basis, or the presence of severe life-threatening disease in the short term.
Ethics oversight	The stool donor cohorts received ethical approval from the regional Committee for the Protection of People (CPP Ouest III) and from the National commission for Protection of Personal data on November 23th 2015. All patients were informed before inclusion and their consent was obtained before analysis.

Note that full information on the approval of the study protocol must also be provided in the manuscript.
